# A novel approach to patients with schizophrenia and type 2 diabetes showing low treatment compliance

**DOI:** 10.1192/j.eurpsy.2022.1983

**Published:** 2022-09-01

**Authors:** P. Argitis, A. Karampas, P. Bringiotti, S. Karavia, O. Pikou, F.-E. Kakavitsas

**Affiliations:** 1 General Hospital of Corfu, Psychiatric, Corfu, Greece; 2 General Hospital of Corfu, General Medicine, corfu, Greece; 3 General Hospital of Corfu, Dermatology, Corfu, Greece

**Keywords:** schizophrénia, Type 2 diabetes

## Abstract

**Introduction:**

Patients with schizophrenia usually demonstrate low compliance to medication. This could be a component of the disorder or a fact that they are not being properly cared.

**Objectives:**

To prevent this in, we tried to treat these patients with long term action antidiabetic agents, in order to achieve better compliance.

**Methods:**

HbA1C measurements of patients suffering from schizophrenia and at the same time receiving oral antidiabetic treatment were conducted. 62 patients were found that fell under the criteria of non regulated type 2 diabetes and at the same time presented less than 70% complied with their antidiabetic pharmaceutical treatment. We modified the antidiabetic treatment of these patients, with the introduction of dulaglutide.

**Results:**

Without intervening with their nutritional habits there was a decline in HbA1C measurements from the average rate of 9,4% to the average rate of 7,6%, as well as an average 6,31% reduction of their body weight.

**Conclusions:**

Due to the improvement of the general medical condition of these patients, the answer to the question whether these patients should be treated with a long term antidiabetic medicines, is positive. The arrival of new long term action antidiabetic medicines in the near future, promises to improve the life quality of schizophrenic patients furthermor.

**Disclosure:**

No significant relationships.

